# The Combined Process of Paper Filtration and Ultrafiltration for the Pretreatment of the Biogas Slurry from Swine Manure

**DOI:** 10.3390/ijerph15091894

**Published:** 2018-08-31

**Authors:** Yuanhang Zhan, Hongmin Dong, Fubin Yin, Caide Yue

**Affiliations:** Institute of Agricultural Environment and Sustainable Development, Chinese Academy of Agricultural Sciences, Beijing 100089, China; zzyh727@126.com (Y.Z.); carft_257@163.com (F.Y.); ycdhope@163.com (C.Y.)

**Keywords:** biogas slurry, the combined process, paper filtration, ultrafiltration

## Abstract

The membrane process had been applied for the advanced treatment of pig farm biogas slurry. As studied, this physical pretreatment, with low cost and high efficiency of the suspended solids removal and nutrient retention, is required to control membrane fouling. The combined process of paper filtration and ultrafiltration in a pilot scale was applied in the present study. The main objective was to explore and identify the feasibility of the new process for the pretreatment of the separation liquid of pig farm biogas slurry. A precision identification experiment of paper filtration and the multi-batch repetitive experiments of the combined process were designed. The results showed, at the identified paper filtration precision of 50μm and an operating pressure of 0.3 bar of the ultrafiltration process, that the flux rate at the stable stage of the multi-batch operation was around 295.00 L/h. The combined process achieved an overall processing rate of 345.41 ± 18.81 L/h and a volume permeation proportion of 82.45% ± 0.85%. The TSS was removed by 95.71%, but total nitrogen (TN) and ammonia nitrogen (NH_3_-N) were retained by 76.29% ± 2.04% and 73.74% ± 2.10%, respectively. Comprehensively, the requirement for the pretreatment was obtained.

## 1. Introduction 

The anaerobic fermentation of pig farm sewage for biogas production is a widely used and highly resource-intensive technology, but a large volume of biogas slurry is produced from biogas plants [1], which are still rich in nutrients and pose the potential benefit for the cultivated land base [2]. A prior study showed that there were more than 73,000 biogas plants with the generation capacity of 3.9 billion tones for biogas slurry in 2011 in China [3]. The biogas slurry, with the large volume and the intricate composition, contains more than 90% water and tremendous number of suspended solids (SS) and organics [4]. Thus, the transport of raw biogas slurry to far-away farms is of high cost and direct land application faces the problems of insufficient consumption, low utilization efficiency and secondary pollution. Membrane process can not only concentrate the slurry in a small volume with enrichment of the nutrients, but also obtain fresh water products [5]. However, the large amount of suspended solids in the biogas slurry may cause membrane fouling and blocking [6], which may reduce the processing efficiency, shorten the service life of the membrane and increase its processing costs. To ensure the long-term efficient membrane processing, pretreatment should be performed to remove most of the SS while retaining most of the nutrients in the biogas slurry.

The typical pretreatment technologies can be divided into three types, according to the principles, which are chemical treatment [5,7,8,9], biological treatment [10,11,12] and physical treatment [2,13,14]. 

Chemical treatment might impact the composition of the biogas slurry, resulting in the influence on the enrichment of the nutrients. Fangueiro et al. [5] reported that acidification resulted in a large loss of mineral components due to the leaching and solidification, thus the fertilizer value, as well as the associated nitrogen, phosphorous, or carbon dynamics changed. Biological treatment will have a long cycle, with complex and difficult conditions to control. Hailing et al. [10] applied immersed MBR, combined with batch aeration in processing anaerobic digesters in pig farms, it had long processing time, and there was an emission of ammonia and other greenhouse gases during the operation. Gravity precipitation, centrifugation and medium filtration (sand filtration, cartridge filtration, multistage filtration, etc.) are the major kinds of physical pretreatment for membrane process. The physical complex multistage filtration might result in great nutrient loss. Longlong et al. [15] found that with the multistage filtration of straw, volcanic rock and quartz sand for the pretreatment of pig farm biogas slurry, the total nitrogen was reduced by 46.5%. Paper filtration was a new choice for pretreatment of the pig farm biogas slurry, which might not only retain the nutrients, but also would not harm to the environment, as the paper cloth could be directly returned to the compost or the field. 

However, single medium filtration often fails to meet the requirements as a pretreatment for nanofiltration (NF) or reverse osmosis (RO), which focus on nutrient concentration. Guorui [16] applied the multistage filtration of multi-layer steel grille, quartz sand filter and security filter for the pretreatment of biogas slurry, the study found that as the filtrate was directly fed into NF, the membrane flux was reduced by 50% after only 50 min. Therefore, microfiltration (MF) and ultrafiltration (UF) membrane separation are usually applied as further pretreatment to remove smaller particles. MF retains particles between 0.1 and 5 mm, while UF removes macromolecules and particles in the 0.001–0.05 mm size range [17]. These treatments were applied for the further removal of SS and macromolecules. Inorganic ceramic membrane and organic hollow fiber ultrafiltration membrane are the two main membrane types in practical use for membrane separation pretreatment, while the hollow fiber ultrafiltration membrane might have a relative higher flux rate and require lower operating pressure than ceramic membrane, as F. Waeger [18] reported. 

The combination of a pretreatment and membrane separation process had been applied in many studies. Caide [19] applied the combination of a Y-strainer and ceramic membrane for the pretreatment of pig farm biogas slurry. Ledda [4] applied the combination of centrifuge separation and ultrafiltration for the digested slurry. Ruan [2] appiled the combination of sand filter, MF and UF for the pretreament of pig farm biogas slurry. The combination of physical pretreatment and membrane separation was proved to have simple operation and few environmental risks and it could remove most of the suspended solid matter while retaining most of the organic matter and N nutrients in the permeate, which could be enriched and reused by NF or RO membrane concentration. 

In this study, the new combined process of paper filtration and hollow fiber membrane ultrafiltration was designed and tested to treat the separation liquid of the pig farm biogas slurry in a pilot scale. In the combined process, the feasible paper filtration precision, the permeate flux rate, the efficiency of the suspended solids removal and nutrient retention and the processing rate were identified. The main objective was to identify the feasibility of the new combined process as a practical pretreatment of the pig farm biogas slurry for nutrient concentration and fresh water production. 

## 2. Materials and Methods 

### 2.1. Biogas Slurry and Sample Testing Methods 

The separated liquid of the biogas slurry from a large-scale pig farm in Hebei Province, China was applied to these experiments. The main composition of the separated liquid is shown in Table 1.

The physico-chemical characterization in this study included pH, electrical conductivity (EC), turbidity, chemical oxygen demand (COD), total nitrogen (TN), ammonia nitrogen (NH_3_-N), total phosphorus (TP), total suspended solids (TSS) and total solids (TS). The reference methods are shown in Table 2. The agents (Chemical Purity Grade) were used as received with no further purification. Deionized water was used throughout all the tests.

### 2.2. Paper Filter and the Ultrafiltration Membrane

The gravity-type paper filter was used for the physical pretreatment of the separation liquid of the pig farm biogas slurry. The filter cloths were made of polypropylene, with the common precision of 15–50 μm and a width of 0.5 m. There was a conveyor belt under the filter cloth powered by a small motor, with a storage tank at the bottom. During the combined process, the slurry was pumped onto the filter cloth; the filtrate flew into the storage tank and the filter cloth was blocked. The liquid level on the filter cloth rose until the float on the liquid surface touched the relay switch, resulting in the automatic replacement of the new filter cloth by the motor operation, after which the liquid level on the filter cloth would drop and the motor would stop. The new filter cloth was automatically replaced until it was clogged. The replaced filter cloth went directly to the compost.

A hollow fiber membrane of ultrafiltration was used after the paper filter. The characteristics of the membranes are shown in Table 3.

**Figure 1:**
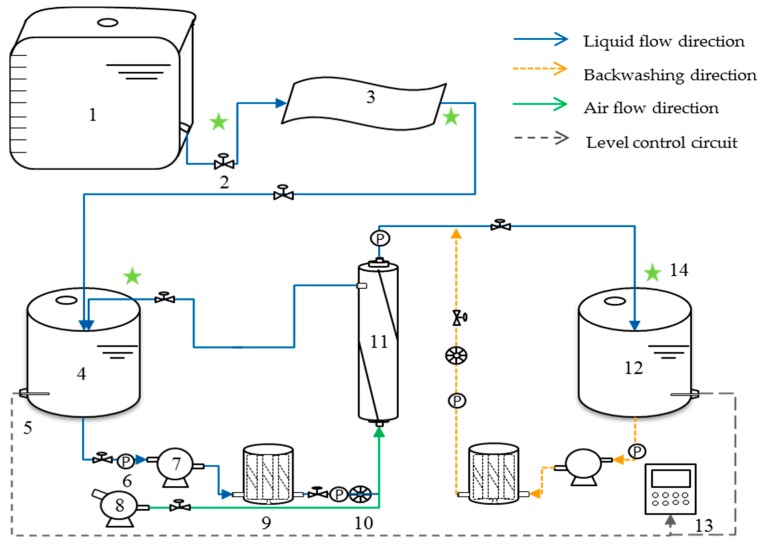
Schematic diagram of the combined process of paper filtration and hollow fiber membrane ultrafiltration for pretreatment of pig farm biogas slurry. 1: Biogas slurry separation liquid storage tank; 2: ball Valve; 3: paper filter; 4: filtrate storage tank; 5: level gauge; 6: pressure gage; 7: vacuum pump; 8: air compressor; 9: bag filter; 10: pressure regulator; 11: hollow fiber ultrafiltration membrane; 12: permeate storage tank; 13: PLC (Programmable Logic Controller) control cabinet; 14: sampling point.

The overall process schematic diagram was shown in Figure 1. The separation liquid of the pig farm biogas slurry was pumped to the cloth of the paper filter and the filtrate was collected in the filtrate storage tank. The filtrate was then pumped from the filtrate storage tank to the membrane by the feed pump and a bag filter was used ahead of the membrane for protection. The ultrafiltration membrane permeate was collected into the permeate storage tank, while the concentrate was circulated to the filtrate storage tank. A short period of clean water washing, together with backwashing, was performed to achieve a better recovery of initial permeate flux after short periods of filtration. Aeration was also performed during the washing, which was reported as an efficient technique to prevent irreversible fouling and maintain high permeation through the membranes [20].

### 2.3. Experimental Procedures

#### 2.3.1. Paper Filter Precision Identification

To identify the appropriate precision of paper filters, the common precision values of 15, 30 and 50 μm were tested. The filter cloths with different filter precisions were fixed on the inlet of the production liquid bucket, then the separation liquid from the original liquid bucket was slowly added to the filter cloth until the filter was blocked and three repeat tests were performed for each precision of the filter cloth. The original liquid bucket and the production liquid bucket had the same specifications, with the inlet diameter of 24.4 cm. The changes of the original liquid depth at different time were measured and recorded to calculate the flux rate of paper filter. The sample of the separation liquid and the filtrate were taken and detected according to methods in Table 2.

#### 2.3.2. The Combined Process Treatment of the Pig Farm Biogas Slurry

Four batches repetitive tests were performed with the influent liquid volume of 450 L and the identified paper filtration precision. The ultrafiltration process was run at an operational pressure of 0.3 bar. The frequency of filter cloth replacement and the filtrate volume were recorded. The volume and flux of the permeate and the concentrate, the inlet and outlet pressure of membrane, the water supply pressure and the liquid temperature were measured every 3 min during the ultrafiltration process. The volume was read by a tank level sensor, the flux was read by the float flow meter, the pressure was read by the pressure gauge and the liquid temperature was read by the temperature sensor. The separated liquids of the biogas slurry, filtrate, permeate, and concentrate that were sampled were detected according to Table 2.

### 2.4. Data Analysis Method

The test data were recorded, calculated, analyzed and plotted using Microsoft Excel 2016 (Microsoft, Redmond, WA, USA). Analysis of variance was analyzed by the independent sample *t* test, with a significance level of 0.05, using IBM SPSS Statistics 20.0 (IBM, Armonk, NY, USA).

Removal rate (Rr) was used to reflect the removal effect of the substance in the permeate. It can be calculated by the following equation [21]:

(1) $$\mathrm{Rr}\left(\%\right)=\frac{C_{i}-C_{p}}{C_{i}}*100$$

where *C_i_* is the concentration or content of the substance in the separation liquid and *C_p_* is the concentration or content of the substance in the permeate.

Initial flux recovery (*FR*) was used to assess the cleaning efficiency of ultrafiltration. It can be calculated by the following equation [22]:

(2) $$FR\left(\%\right)=\frac{F_{w}-F_{fw}}{F_{jw}-F_{fw}}*100$$

where *F_w_* is the initial flux rate (L/h) after cleaning, *F_fw_* is the flux rate (L/h) after membrane fouling and *F_jw_* is the flux rate (L/h) before membrane fouling.

The average permeate flux rate (PF) was used to assess the production efficiency. It can be calculated by the following equation:

(3) $$\mathrm{PF}\left(L/h\right)=\frac{V_{p}}{t_{b}}$$

where *V_p_* is the permeate liquid volume (L) and *t_b_* is the batch processing time (h).

A concentration factor (CF) was used to assess the concentration effect of the substance in the concentrate. It can be calculated by the following equation [23]:

(4) $$\mathrm{CF}=\frac{C_{c}}{C_{i}}$$

where *C_c_* is the concentration or content of the substance in the concentrate and *C_i_* is the concentration or content of the substance in the separation liquid.

## 3. Results

### 3.1. Precision of Paper Filtration

**Figure 2:**
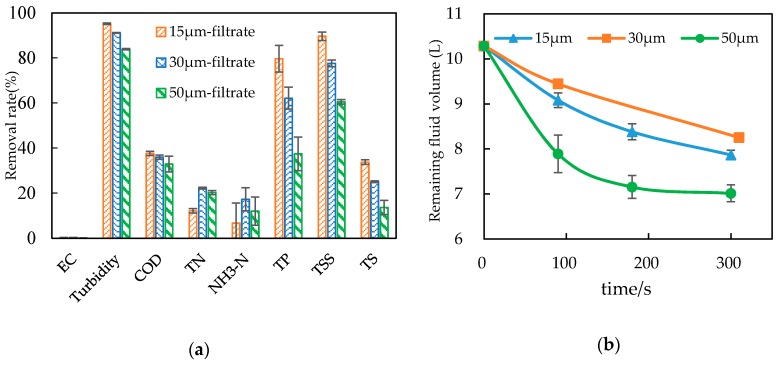
Water parameters removal rates (**a**) and variations in volume of separation liquids (**b**) by filter cloths with different precisions.

The paper filter cloths with different filter precisions (15, 30 or 50 μm) had significant differences in the removal rates of turbidity, TP, COD, TSS and TS, as shown in Table 4 and Figure 2a; among these, turbidity and TSS had good removal effects, with turbidity removal rates for each filter precision level of 95.15% ± 0.94%, 91.14% ± 0.96% and 83.91% ± 3.49% and TSS removal rates of 89.64% ± 1.05%, 77.59% ± 0.40% and 60.50% ± 3.14%, respectively. The COD removal rates were 37.61% ± 1.02%, 35.93% ± 0.40% and 32.88% ± 0.87% respectively, while the removal rates of EC, TN and NH_3_-N were low; there were no significant differences among filter cloths with different filter precisions, as shown in Table 4 and Figure 2b. The paper filtration pretreatment was designed to remove the macromolecular organics and TSS and according to the membrane technical manual and related studies [18,24,25,26], the paper filtration filtrates with the filtering precision of 15, 30 and 50 μm could all meet the requirements for the influent liquid of the hollow fiber ultrafiltration. The filtration rate was the fastest at the filtration precision of 50 μm, as shown in Figure 2b, which means a high efficiency and a relatively low cost of electric energy and filter cloth. Thus, a precision of 50 μm for the paper filter filtration is recommended. 

### 3.2. The Combined Process of Optimized Paper Filtration and Ultrafiltration

#### 3.2.1. Flux Rate of the Combined Process

**Figure 3:**
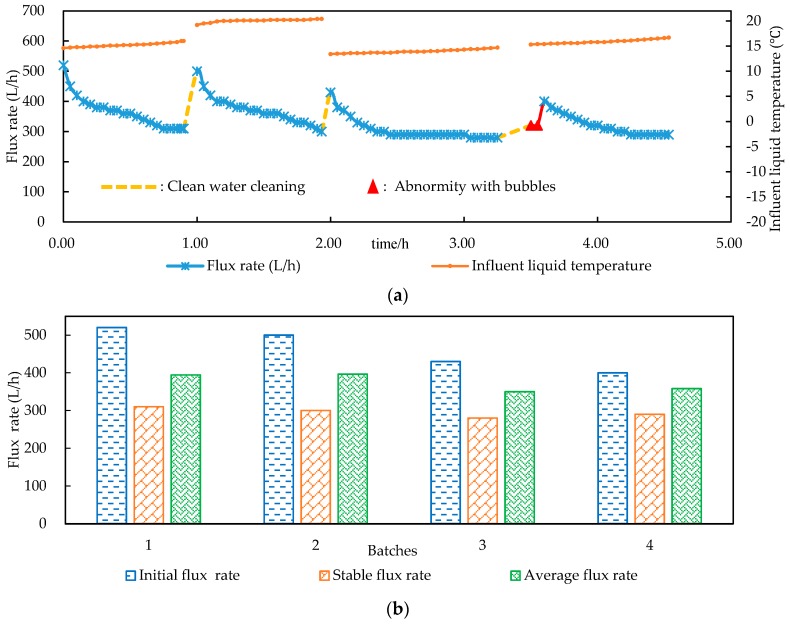
Variations (**a**) and stability analysis (**b**) in the flux rate of multi-batch ultrafiltration process.

The flux rate of the combined process varied with the batch order and the running time, as shown in Figure 3. There were two stages in the variations of the flux rate in each single batch operation. The flux rate rapidly decayed with time at the first stage, which could be related to the fact that more and more foulants were deposited on the surface of the membrane and the membrane pores were blocked, resulting in the dramatic decrease of permeation flux. However, the flux rate stayed steady at a certain value at the stable end stage, which could be related to the compaction and thickening of the fouling cake layer.

At the end of each batch, washing and backwashing for the UF membrane were carried out. The initial flux rate of each batch was restored, as shown in Figure 3. The PFs of the four batches were 394.44 L/h, 396.43 L/h, 350.00 L/h and 358.06 L/h, respectively. The FRs were 90.48%, 65.00% and 80.00% in the second, third and fourth batches, respectively. The liquid temperature was relatively higher in the second batch, as shown in Figure 3a. Studies [24,27] had reported that liquid temperature affected the rate of molecular permeate through the membrane, so the flux rate would be higher as the liquid temperature increased. The FR in the third batch was thus relatively low, as the decline of the flux rate was relatively high in the second batch, according to the equation 2. The flux rates at the stable stage of the four batches were 310.00 L/h, 300.00 L/h, 280.00 L/h and 290.00 L/h, respectively. The attenuation was very small, indicating that the flux rate in the steady stage of each batch remained basically stable. At an influent liquid volume of 450 L for each batch, the average processing rate of the paper filtration was about 3.27 times that of the hollow fiber ultrafiltration and the processing rate of the integrated process was 345.41 ± 18.81 L/h.

#### 3.2.2. Effects of the Combined Process on Water Quality

The qualities of the permeate and concentrate from the combined process is shown in Table 5, where the mean and standard deviation value are presented. Compared with the initial separation liquid (Table 1), the qualities of the permeate (Table 5) showed no differences of EC and pH and the Rr of the EC was 0.83% ± 2.39%, which was close to 0. The TN and NH_3_-N did not decrease significantly with the low Rrs of 7.51% ± 3.48% and 10.62% ± 3.18%, respectively. COD and TSS decreased in a certain extent with Rrs of 38.78% ± 3.16% and 37.63% ± 1.23%, respectively. The TP, turbidity and TSS were significantly reduced, with Rrs of 81.01% ± 3.23%, 95.71% ± 1.35%, 95.62% ± 0.90%, respectively. The treatment of the combined process of paper filtration and hollow fiber ultrafiltration had a significant removal of turbidity and TSS and the removal rate could reach over 95%. 

**Figure 4:**
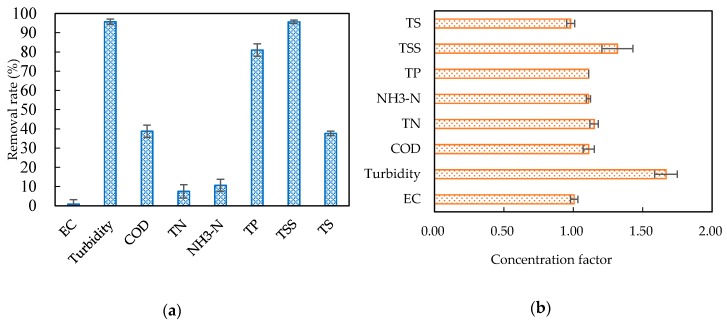
The removal rates in the permeate (**a**) and the concentration factors in the concentrate (**b**), compared with the initial separation liquid.

Compared with the initial separation liquid (Table1), the concentrate of the combined process had no effect on the concentration of EC and TS, as shown in Figure 4b; the CFs of COD, NH_3_-N, TN, TP and TS were between 1.00 and 1.20, as shown in Figure 4b, indicating a weak enrichment effect. The CFs of TSS and turbidity were 1.33 ± 0.11 and 1.67 ± 0.08, respectively, as shown in Figure 4b, indicating a certain enrichment effect. 

#### 3.2.3. The Substance Flow and Distribution during the Combined Process

The substance flow and distribution of various components in the separation liquid during the combined process were calculated and analyzed. The mean and standard deviation values were presented in Table 6. The volume permeation proportion was 82.45% ± 0.85%, as shown in Table 6. Compared with the initial separation liquid, most of the TN and NH_3_-N was retained in the permeate in the proportions of 76.29% ± 2.04% and 73.74% ± 2.10%, respectively. The proportions of TS and COD distributed in the permeate were slightly lower, at 51.48% ± 1.46% and 50.49% ± 2.07%, respectively, while the proportion of TP was 15.54% ± 2.53%. Only 3.60% ± 0.70% of TSS was distributed in the permeate. The proportions of the substances distributed in the concentrate, including COD, TN, NH_3_-N, TP, TSS and TS, were close to the volume proportion, which was 12.83% ± 0.79%. 

## 4. Discussion

The results in this study showed that the combined process had an overall processing rate of 345.41 ± 18.81 L/h and a volume permeation proportion of 82.45% ± 0.85%, at a working pressure of 0.3 bar. In the study of Caide [19], which applied Y-strainer and ceramic membrane for the pretreatment of the pig farm biogas slurry, the operating time was about 3 hours; at a working pressure of 3 bar and a batch volume of 300 liters, over 99% of TSS was removed and the retention rates of the TN and NH_3_-N concentrations were over 90% in the permeate. Ledda [4] applied the combination of centrifuge separation and ultrafiltration for the pretreatment of the digested slurry. That study found that in the permeate, TN decreased from 45.7 ± 1.5 kg/cycle to 11 kg/cycle and NH_3_-N decreased from 22.5 ± 0.3 kg/cycle to 10.6 kg/cycle. Ruan [2] appiled the combination of a sand filter, MF and UF for the pretreament of pig farm biogas slurry; it was reported that the operating time was about 1 hour at a batch volume of 200 liters and in the permeate, NH_3_-N concentration deceased from 984 mg/L to 927 mg/L. Compared with the previous literature, the new combined process in this study had a relatively high processing efficiency and low operation pressure and most of the SS was removed while most of the N nutrients were retained. The comparisons showed that this combined process might be a new practical choice for the pretreatment of the pig farm biogas slurry.

The abnormity with bubbles in the fourth batch, which was noticed in the effluent liquid from the float flow meter, might be explained by the formation of the fouling cake layer on the membrane. It was reported that membrane fouling accumulated on the surface of UF membrane as the slurry was filtrated [17]. Air was left in the fouling cake layer because of the liquid evacuation during the cleaning process, until the layer got thick enough that the air left inside could not be evacuated all at once, but had to be slowly be discharged by the influent liquid. The bubbles were thus noticed from the float flow meter, and the initial flux rate was read much lower. The flux rate had an obvious recovery after the air was evacuated and there were no bubbles noticed in the float flow meter. A long period of abnormity with bubbles might indicate problem of membrane fouling and necessity for membrane cleaning.

According to the mass balance during the combined process and Table 6, some substances in the initial separation liquid were rejected and left on the filter cloth, including most of the TSS with the proportion of 59.96%; some of the COD, TP and TS, with the proportion of 24.70%, 39.17% and 29.16%, respectively; and only 9.07% of TN and 7.56% of NH_3_-N. The substance left could go directly to the compost with the filter cloths. Some substances in the filtrate were also left in the membrane system and the proportions of COD, TN, NH_3_-N, TP, TSS and TS were 13.57%, 0.66%, 4.98%, 35.86%, 21.12% and 10.06%, respectively. These parts of the substance might be left in the froth or sediment in the filtrate storage tank, which might be returned to the biogas slurry pool together with the concentrate. In addition, the low retention of TP in the permeate might be related to the phosphorus solidification—it was reported that phosphorus was predominantly linked to particles between 0.45 and 10 mm [28]. 

The results in this study show that the concentrate contained slightly high concentrations of organics, nutrients and TSS; however, it was not recommended to make fertilizer, as the distributed volume proportion was low and the concentration factors of the nutrients were much lower than the NF or RO concentration as reported. The study reports that UF systems are typically not used for concentrating soluble elements [17]. Therefore, the concentrate of the combined process was recommended to be returned to the biogas slurry pool.

The water quality of the permeate, with a volume permeation proportion of 82.45% ± 0.85% by the combined process, could meet the requirement for the influent liquid of the NF membrane process [29,30]. Therefore, the permeate would go to the NF system for further water purification and nutrient concentration.

The bubbles in the effluent liquid and the decline of the initial flux rate in the combined process indicated that UF membrane fouling have a great influence on the processing efficiency and cost in the long term operation, membrane cleaning would also be necessary to eliminate the membrane fouling [17]. To further identify the practical application of the combined process, assessment of the processing efficiency and cost based on long term operation should be carried out. The assessment might include the stability of flux rate and water quality, the operation cost, the membrane cleaning strategy and its effectiveness and the replacement frequency of the paper filter cloth, etc.

## 5. Conclusions

Based on the study, paper filtration with the precision of 50 μm, combined with the hollow fiber membrane ultrafiltration, was recommended as the physical pretreatment of the separation liquid of the pig farm biogas slurry. In the multi-batch operation at the ultrafiltration operating pressure of 0.3 bar, the flux rate at the stable stage was around 295.00 L/h. The combined process achieved an overall processing rate of 345.41 ± 18.81 L/h and a volume permeation proportion of 82.45% ± 0.85%. Over 95% TSS was removed, but most of TN and NH_3_-N were retained by 76.29% ± 2.04% and 73.74% ± 2.10%, respectively, indicating that the requirements were achieved as the pretreatment of nanofiltration or reverse osmosis concentration. Based on these results, further assessment of the efficiency and cost based on long term operation were advised for practical application.

## Figures and Tables

**Table 1:** Composition of the biogas slurry solid-liquid separation liquid.

Component	Content (Means ± SD)
pH	7.11 ± 0.23
EC (ms/cm)	7.23 ± 0.16
Turbidity	279.00 ± 29.88
COD (mg/L)	852.33 ± 48.35
TN (mg/L)	773.33 ± 18.86
NH_3_-N (mg/L)	684.67 ± 15.17
TP (mg/L)	40.65 ± 3.25
TSS (mg/L)	628.00 ± 68.82
TS (mg/L)	2889.33 ± 54.09

**Table 2:** The summary of physico-chemical analysis methods considered in this study.

Component	Reference Methods
pH	Electrometric method (Five Go F2, Switzerland)
EC (ms/cm)	Determination of conductivity ((Five Go F3, Switzerland)
Turbidity	Nephelometric method (2100 P Hach, America)
COD (mg/L)	Potassium dichromate method (HJ/T 399-2007, China)
TN (mg/L)	Potassium persulfate oxidation method (HJ 636-2012, China)
NH_3_-N (mg/L)	Salicylic acid hypochlorite spectrophotometry method (HJ 536-2009, China)
TP (mg/L)	Ammonium molybdate spectrophotometric method (GB/T 11893-1989, China)
TSS (mg/L)	Total suspended solids dried at 103–105 °C (GB/T 11901-1989, China)
TS (mg/L)	Total Solids dried at 103–105 °C (GB/T 5750-2006, China)

**Table 3:** Characteristics of hollow fiber ultrafiltration membranes.

Parameter	Characteristic
Material	Polyvinylidene fluoride (PVDF)
Surface Material	Rigid Polyvinyl chloride
Pore feature (nm)	10–100
Filtration area (m^2^)	30
Filtration mode	Pressure Screening
Max. pressure (bar)	3
Max. temperature (°C)	45
pH range	2–10

**Table 4:** The significance of water parameter removal rates (%) in filtrates by filter cloth with different filtering precisions.

Water Parameters	EC	Turbidity	COD	TN	NH_3_-N	TP	TSS	TS
*p*-value	0.542	0.006	0.003	0.369	0.303	0.001	0.000	0.001

**Table 5:** The water parameters of the paper filtration filtrate, the ultrafiltration concentrate and the ultrafiltration permeate (mean ± SD).

Water Parameters	pH	EC (ms/cm)	Turbidity (NTU)	COD (mg/L)	TN (mg/L)	NH_3_-N (mg/L)	TP (mg/L)	TSS (mg/L)	TS (mg/L)
The filtrate	7.10 ± 0.10	7.20 ± 0.18	33.03 ± 7.48	658.67 ± 43.02	721.67 ± 16.50	649.67 ± 10.96	25.41 ± 1.63	193.33 ± 18.57	2101.33 ± 64.86
The ultrafiltration concentrate	7.24 ± 0.13	7.24 ± 0.05	54.50 ± 9.85	731.67 ± 24.39	830.00 ± 14.14	720.33 ± 7.32	28.25 ± 2.24	254.67 ± 4.99	2061.33 ± 52.80
The ultrafiltration permeate	7.19 ± 0.08	7.17 ± 0.07	11.56 ± 2.49	520.33 ± 9.98	715.00 ± 25.50	611.50 ± 8.03	7.83 ± 1.93	28.00 ± 8.64	1801.33 ± 1.89

**Table 6:** The substance flow and distribution of various components in the liquid during the combined process (mean ± SD).

The Substance Distribution	COD	TN	NH_3_-N	TP	TSS	TS	Volume (L)
The initial liquid (g)	382.6 ± 30.9	346.47 ± 2.33	306.84 ± 7.48	18.03 ± 1.95	281.34 ± 30.61	1294.75 ± 19.8	448.75 ± 11.39
The filtrate (g)	288.14 ± 26.49	315.03 ± 8.61	283.55 ± 3.55	10.94 ± 0.95	84.29 ± 7.22	917.47 ± 35.76	436.25 ± 10.83
The concentrate (g)	42.66 ± 1.91	48.43 ± 2.47	42.04 ± 2.11	1.7 ± 0.13	14.87 ± 0.87	120.36 ± 7.73	57.50 ± 2.50
The permeate (g)	192.6 ± 9.14	264.33 ± 6.94	226.29 ± 8.98	2.85 ± 0.76	10.34 ± 3.17	666.51 ± 22.68	370.00 ± 10.61
The proportions in the filtrate (%)	75.3 ± 3.45	90.93 ± 2.38	92.44 ± 1.27	60.83 ± 1.28	30.04 ± 0.77	70.84 ± 1.69	97.22 ± 0.54
The proportions in the concentrate (%)	11.24 ± 1.13	13.98 ± 0.8	13.72 ± 1.01	9.43 ± 0.27	5.32 ± 0.4	9.3 ± 0.65	12.83 ± 0.79
The proportions in the permeate (%)	50.49 ± 2.07	76.29 ± 2.04	73.74 ± 2.1	15.54 ± 2.53	3.6 ± 0.7	51.48 ± 1.46	82.45 ± 0.85
